# LiDAR-OSM-Based Vehicle Localization in GPS-Denied Environments by Using Constrained Particle Filter

**DOI:** 10.3390/s22145206

**Published:** 2022-07-12

**Authors:** Mahdi Elhousni, Ziming Zhang, Xinming Huang

**Affiliations:** Department of Electrical and Computer Engineering, Worcester Polytechnic Institute, Worcester, MA 01609, USA; zzhang15@wpi.edu (Z.Z.); xhuang@wpi.edu (X.H.)

**Keywords:** LiDAR, OSM, cross-modal localization, particle filter

## Abstract

Cross-modal vehicle localization is an important task for automated driving systems. This research proposes a novel approach based on LiDAR point clouds and OpenStreetMaps (OSM) via a constrained particle filter, which significantly improves the vehicle localization accuracy. The OSM modality provides not only a platform to generate simulated point cloud images, but also geometrical constraints (e.g., roads) to improve the particle filter’s final result. The proposed approach is deterministic without any learning component or need for labelled data. Evaluated by using the KITTI dataset, it achieves accurate vehicle pose tracking with a position error of less than 3 m when considering the mean error across all the sequences. This method shows state-of-the-art accuracy when compared with the existing methods based on OSM or satellite maps.

## 1. Introduction

Localization is one of the key modules in an autonomous driving system. Knowing the precise location of the vehicle is critically important to perception and controls. LiDAR sensors have been proven to be very useful when attempting to solve localization challenges [1,2,3,4], owing to their accurate and dense representations of the vehicle’s surroundings. The localization results achieved by LiDARs can be further enhanced based on pre-built 3D maps [5,6]. These 3D maps, sometimes called HD maps, are built by concatenating successive point clouds that were aligned by using additional data from other sensors (e.g., differential GPS), or by using a complex SLAM pipeline, which would typically include practices such as loop closure. Then the HD maps need to undergo a labelling process, either manually or by using an automated process [7].

Although these types of maps can help achieve a high-level accuracy when tracking the vehicle, they nonetheless come with drawbacks which make their deployment very challenging, due to the current technological issues. For instance,

- Expensive and time-consuming: Building and maintaining such maps requires us to drive repeatedly around all the areas where we would be interested in locating the vehicles, which not only can be tedious, but also very expensive.
- Large storage: HD maps are known to be very large due to the millions of points that they contain, which can also make their deployment difficult in real time.

Therefore, we argue that 3D point clouds may not be the best format to use in terms of maps. In contrast, we propose to combine LiDAR with a widely available, free, and routinely updated map namely OpenStreetMaps (OSM) [8].

**Motivation.** LiDAR sensors excel at representing the geometrical features of the surrounding environment. In the case of a vehicle driving in an urban setting, the surrounding environment is mostly composed of the road and buildings (nearby objects such as cars are included as well), due to the effects of occlusion. Naturally, these two classes are commonly labeled in OSM, and an intuitive way to use them would be to match them across modalities in order to localize a vehicle. Although some impressive advances were made in neural network alignment between camera and LiDAR such as [9,10], matching RGB images extracted from an abstract map such as OSM with point clouds as accurately as possible for localization is very challenging. To address this issue, we propose to use ray casting, combined with a buildings mask that we extract from OSM, so that we can produce simulated LiDAR images. We also extract road masks from OSM and use them in two different ways: (1) as a secondary map where we will localize vehicles on the road extracted from the input point clouds; (2) as the constraint map to make sure that the final localization result does not leave the road, in case of a noisy sensor input.

**Objectives.** Our goal in this method is to propose a non-learning, simple and stable method capable of localizing vehicles in OSM by only using LiDAR senors, with an accuracy level on par with or superior to GPS sensors (which is reported to be more than 5 m in an urban setting [11,12]). This means that our method will be able to replace GPS, or act as backup when GPS is not available. It can also be used as an initial guess to other more advanced sensor suites.

**Approach.** The difficulty of aligning LiDAR point clouds with OSM is mainly owing to the modality gap between these two representations. Point clouds returned by a LiDAR are typically represented by an (*N*, 4) unsorted array representing the (*x*, *y*, *z*) 3D locations and the reflectivity *r* of each point. In contrast, the OSM representation that we are using is an (*L*, *W*, 3) RGB image, representing a top view of the environment, with different classes labeled accordingly.

A common first step when attempting to localize 3D point clouds in 2D aerial maps, is to proceed to a bird’s eye view (BEV) projection, which means first cropping the point cloud to an acceptable region of interest, then projecting it on the ground plane, and re-scaling it according to a pre-defined scale (in this case, matching the scale from OSM), so that it can be represented as a 2D image, where each non-zero pixel stores the height value of the corresponding point. This results in a top view representation of the LiDAR point cloud, and a closer visual appearance to the top view 2D map. However, even after proceeding with this projection, the modality gap is still too big to attempt any direct matching between these two. In order to solve this issue, we propose to first extract a road and buildings mask from OSM, because those are the most common classes of objects in an urban environment. These masks are then used in combination with ray casting, to generate simulated top view LiDAR images. We use a dual input particle filter that attempts to align the simulated LiDAR images with input point cloud BEV images. Because it is reasonable to assume that our vehicle should never leave the road, we also use the road mask to constrain the particle filter, making sure that the final solution always lies within the constrained region.

**Figure 1:**
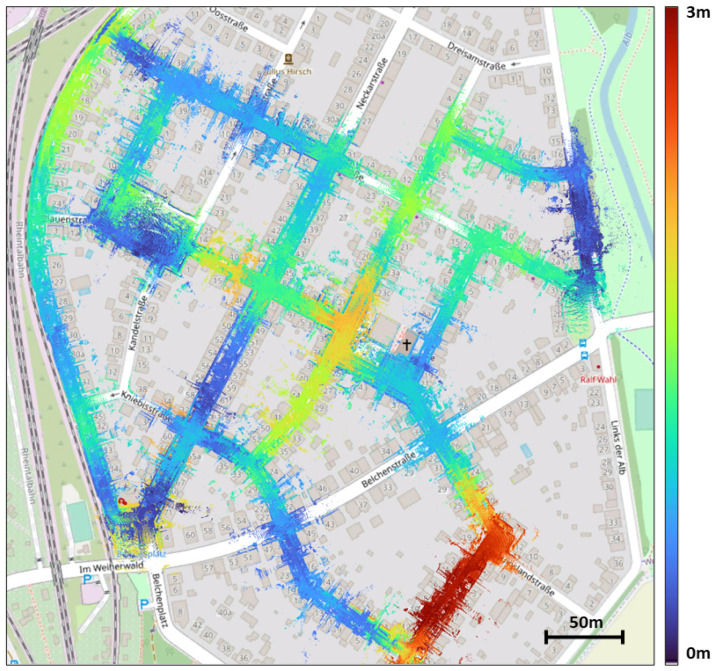
Result of our approach. LiDAR point clouds overlaid on top on OSM. Colors reflects the position error (m).

Our method does not rely on any type of machine learning and uses only open-source maps for LiDAR based localization input. It has the potential to be used anywhere as long as OSM is available, and can act as a replacement of GPS when the GPS sensor or GPS signal is unavailable. Figure 1 shows an example of our method’s results.

**Contributions.** The contributions of this research can be summarized as follows:

- We propose a fast and consistent method to generate simulated top view LiDAR images from OSM, and accordingly show how we can use those images to accurately localize LiDAR point clouds in OSM.
- We propose a dual-input particle filter algorithm with an added constraint that the vehicle location must be on the road.
- We demonstrate the state-of-the-art accuracy of our method on the KITTI dataset, by comparing it to other LiDAR cross-modal localization methods by using OSM or satellite maps.

## 2. Previous Work

### 2.1. Cross-Modal LiDAR Localization

LiDAR localization [13], odometry, and SLAM are classical robotics challenges that have become popular with the advent of autonomous driving, owing to the richness of the data that the sensor produces. Lately, cross-modal LiDAR localization has been attracting more attention, due to the fact that it requires a significant amount of time, effort and resources when trying to build and maintain HD point cloud maps. This led researchers to look for alternative platforms that could be used as maps when trying to localize a vehicle driving in an urban environment. The most accessible and available maps to the public today are top-view 2D maps such as OSM and satellite maps. OpenStreetMaps (OSM) are one type of such maps, which provide 2D shapes of most major structures, tagged with geo-localization data. Thanks to its open-source character, OSM has been very popular within the SLAM research community. In [14], a classifier is trained to distinguish between LiDAR points that fall on the road or not. Based on this information, a cost function is optimized, and a particle filter is used to localize the vehicle in OSM. Another approach was proposed in [15], and is based on matching building planes from LiDAR to cuboids in OSM. In [16], a handcrafted feature descriptor, based on buildings and intersections positions is proposed for global localization in OSM. Moreover, several other works attempted to solve the cross-modal localization task on OSM by using cameras rigs instead of LiDAR sensors, such as [17,18,19].

Another type of top view 2D maps are satellite maps. These maps provide real-world RGB images captured from an aerial position. In [20], the authors leverage semantic segmentation results from both the LiDAR point cloud and the satellite images in order to optimize the soft cost function of a particle filter. In [21], another particle filter is combined with a similarity network, which was trained to match point clouds that were projected to BEV with satellite map crops. In [22] and [23], a generative adversarial network [24] is trained to generate synthetic top-view LiDAR images based on input satellite crops. The synthetic and real LiDAR images are then both fed to a neural network to predict the value of the displacement between frames. In [25], the authors take advantage of the reflectivity field returned by the LiDAR sensor to match their top view projected scans to aerial imagery maps by using the normalized mutual information (NMI) technique [26]. Although these methods can be seen as “dense” methods because they use most, if not all, the points available to them, other methods, such as [27] try to extract and match features (represented by cropped patches) from both modalities by using a similarity-trained CNN. Although satellite maps might provide a more lifelike image of the surrounding environment, they tend to be cluttered with additional objects that are irrelevant, and sometimes detrimental to the localization results, such as cars and trees. That is the main reason why we chose to use OSM instead, which provides clear boundaries and edges of buildings and roads that our method can rely upon.

### 2.2. Constrained Particle Filters

Particle filter (PF) [28] is a sampling-based method which computes the weights of a set of hypotheses based on an observation and motion model, with a final result consisting of a weighted sum of the previously emitted hypotheses. Particle filters, also called Monte Carlo localization (MCL) [29], are very popular in the robotics field and have been used to solve localization challenges for both indoor [30] and large-scale outdoor scenarios [31]. A localization algorithm such as particle filters, owing to the context in which it is applied (where map information is available), does not suffer as much from drifting problems as some other odometry estimation methods. However, noisy observations and inaccurate motion estimation sometimes can lead to large errors.

A natural improvement to the PF would be to use external constraints to limit the final result within a “feasible” region and thus reduce the error produced by the final weighted result [32]. This is in part why constrained particle filters (CPF) were proposed, leading to multiple strategies. For instance, constraints were applied equally to all the particles in a straightforward manner, named the acceptance/rejection approach [33,34], where particles that did not respect the constraints were simply discarded. Other methods such as [35] proposed a complex sampling scheme to only draw particles from the feasible region. Later methods focused on applying the constraints to the final weighted result rather than each hypothetical particle. In [36], the authors propose the mean density truncation approach, where an iterative sampling process gets rid of bad particles and samples good ones, thus gradually pushing the final position to the correct region. Improvements to the sampling process and computation cost were subsequently proposed in [37].

## 3. Method

**Figure 2:**
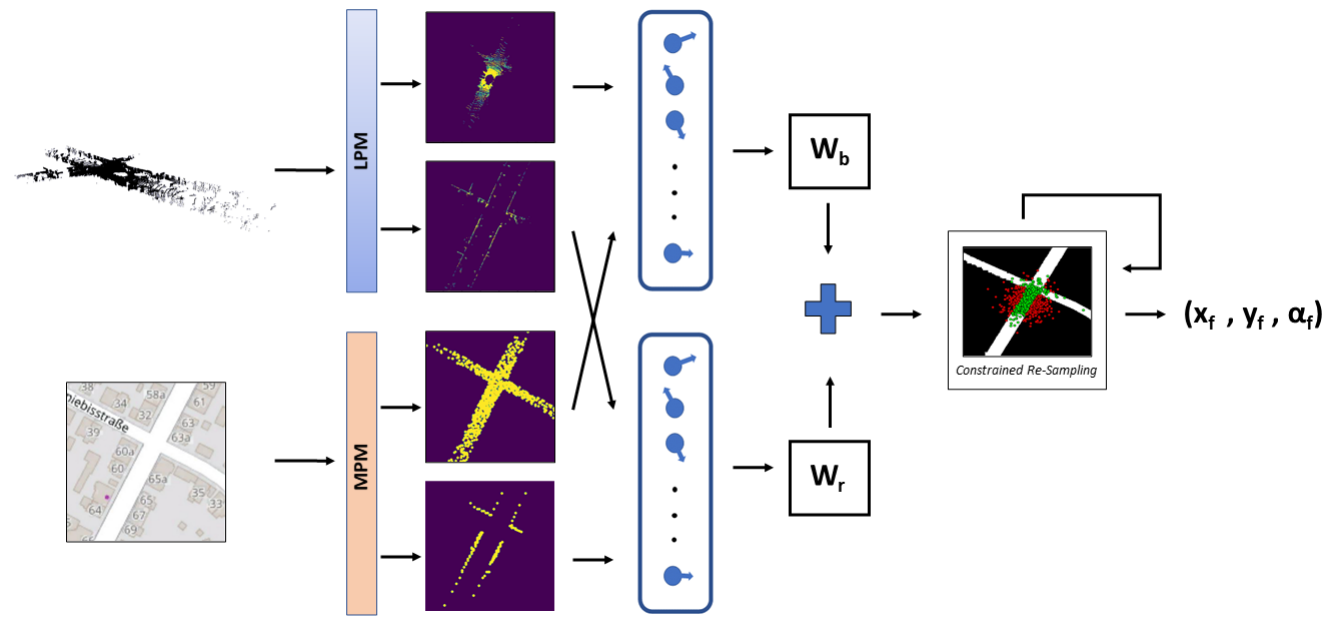
Our full method. The LiDAR point cloud and OSM region of interest are processed by the LiDar processing module (LPM) and map processing module (MPM), respectively, to produce four images, a pair of top-view road images and a pair of top-view building edges, with each pair containing a real and a simulated point cloud image. The two pair of images are processed by a dual input particle filter which produces a first estimate of the vehicle position, followed by a road check to verify if the estimated position is on the road or not. In the latter case, the constrained resampling is triggered, until the road check condition is satisfied.

Our goal is to propose a non-learning and reliable method that can be used to achieve accurate LiDAR 2D pose tracking in OSM. In this section, we first explain how to solve the cross-modality issue for comparing OSM and LiDAR point clouds, then we tackle the pose tracking task by using a road-constrained and dual input particle filter. Figure 2 shows an overview of our proposed method.

### 3.1. From OSM to LiDAR

**Challenges.** The biggest challenge when attempting to solve this task is to bridge the modality gap between unsorted LiDAR point clouds and RGB images from OSM. The most common feature between these two representations is the fact that they both contain geometric information on the shapes of the structures that form the surrounding environment of the vehicle, namely roads, buildings, and sometimes trees. On the other hand, the biggest difference is how both formats represent the data: point clouds are unsorted arrays containing the location information, in a predefined frame, of any point that was reached by the LiDAR sensor, whereas OSM visual maps are top-view RGB images containing rough shapes and class data.

The typical first step when attempting to localize LiDAR point clouds that were collected by a vehicle in a 2D aerial map is to start with a BEV projection, which displays the point cloud data from a top view, giving us a more similar visual appearance to the 2D top-view maps, and thus a first step toward breaching the cross-modality. Unfortunately, this step alone is not enough to accurately localize the vehicle in OSM, mainly due to the sparsity of the BEV image and the effects of occlusion of the point cloud data. This is why we need to use the data provided by OSM, namely the roads and buildings, to produce a simulated LiDAR image, which has similar characteristics as the input LiDAR BEV image.

**Road and Building Masks.** Two of the most clearly labeled classes of objects in OSM are buildings and roads. This information is valuable because those are the classes that are the most present in the point clouds produced by LiDAR sensors when traveling in an urban environment.

**Figure 3:**
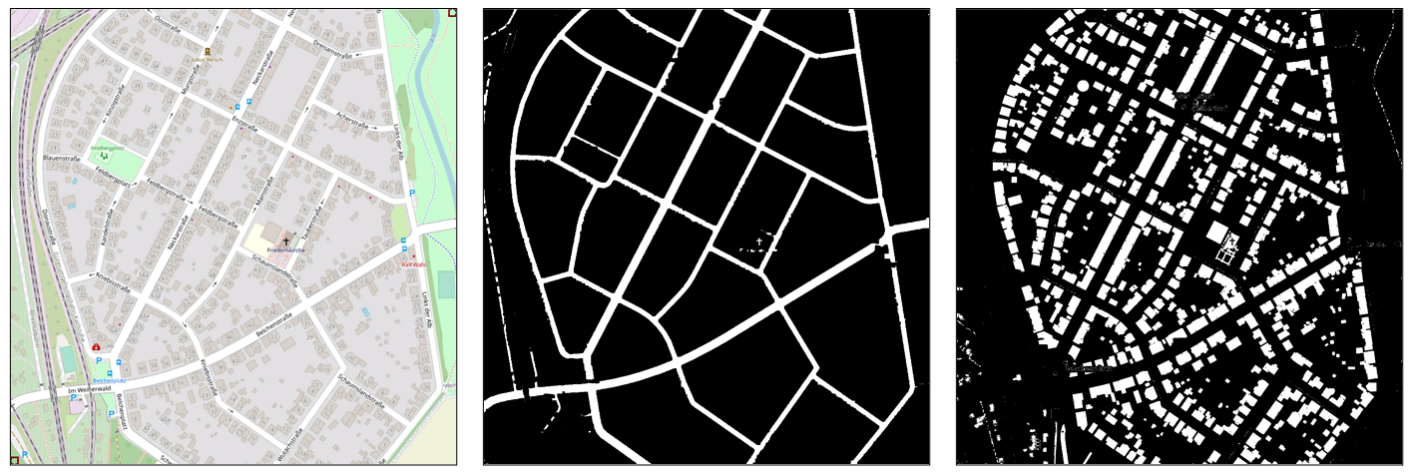
Road and building masks, extracted from OSM.

By using color segmentation (mostly solid white for roads and a specific shade of grey for buildings), combined with morphological transformations such as dilation and erosion, we are able to generate buildings and road masks that we can later use to generate simulated LiDAR top-view images. When extracting the roads, we use the standard OSM layer style. However, for the buildings, we use the public transport layer, to avoid noise resulting from building numbers and business names. The morphological transformations are mostly applied to the road mask, in order to close holes caused by overlaid street names on the images, with a kernel size (5,5) for the dilation and (3,3) for the erosion. It is possible generate our own layers with styles that do not include any overlaid text, but that is out of the scope of this work, and we would rather use already available data. The resulting roads and buildings masks of an area in OSM are shown in Figure 3.

**Simulated and Real LiDAR images.** Before attempting to localize the vehicle, our method first starts by generating two pairs of images: a pair of road top-view point cloud images, and a pair of building edges, also from the top view. Both pairs share an important characteristic: they contain an image generated by using the true LiDAR input point cloud, and a simulated LiDAR image generated by using either the road or building masks, previously extracted from OSM. Next, we will explain how the true LiDAR images are generated, in what we call the LiDAR processing module (LPM), followed by how the simulated LiDAR images are generated, in the map processing module (MPM).

**Figure 4:**
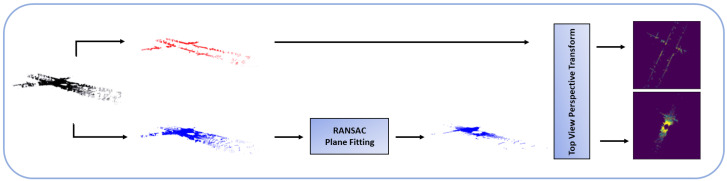
LPM. The LiDAR point cloud is divided into two sections using the height value of each point, a top section (capturing surrounding buildings walls) and bottom one (capturing the road). The bottom section undergoes RANSAC plane fitting to extract the road, then the two point clouds are projected to produce two top-view point cloud images.

In the LPM, we define $\left(x_{L},y_{L},z_{L}\right)$, the frame attached to the LiDAR sensor, with the $z_{L}$ axis pointing upward. The LiDAR sensor itself is fixed to the roof of the car, which makes the $\left(x_{L},y_{L}\right)$ and ground planes parallel to each other. We first start by splitting our input LiDAR point cloud in two by using the height values $z_{Li}$ of each point: a top section, meant to capture the edges of the buildings reached by the LiDAR sensor, and a bottom section, which after being filtered by using RANSAC plane fitting [38], represents the road on top of which the vehicle is traveling. The two point clouds are then projected onto the $\left(x_{L},y_{L}\right)$ plane to generate two BEV LiDAR images. The LPM can be seen in Figure 4.

In the MPM, for the simulated road point cloud, we apply rejection sampling on the road mask with a Gaussian proposal, centered on the supposed vehicle location in OSM (according to the initial guess of the starting position or the previous frame results), and limited to a predefined region of interest. The sampling here is done for two main reasons: first, to simulate the point distribution returned by the LiDAR sensor, which is typically denser around the center of the scan, and becomes sparser the more we move away from the sensor, and second to improve the speed processing, because using all non-zero pixel values in the later calculations would result in a significant slow-down of the whole method.

**Figure 5:**
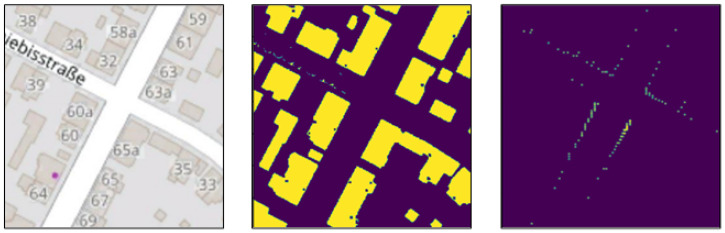
Steps of the raycasting process applied to OSM.

**Figure 6:**
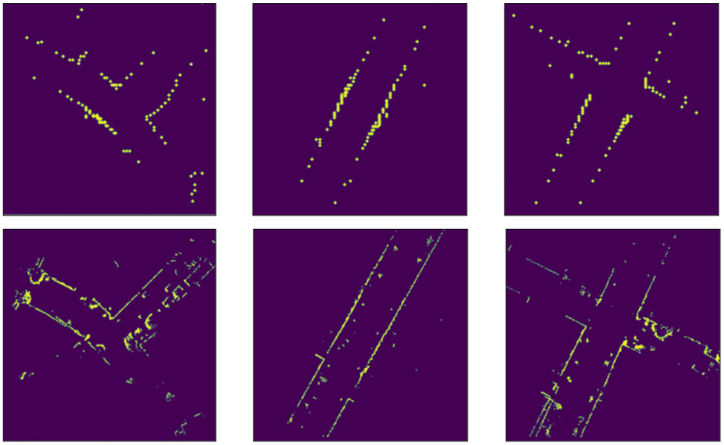
Comparison between LiDAR building images (**top**) and simulated LiDAR images by using raycasting (**bottom**).

**Figure 7:**
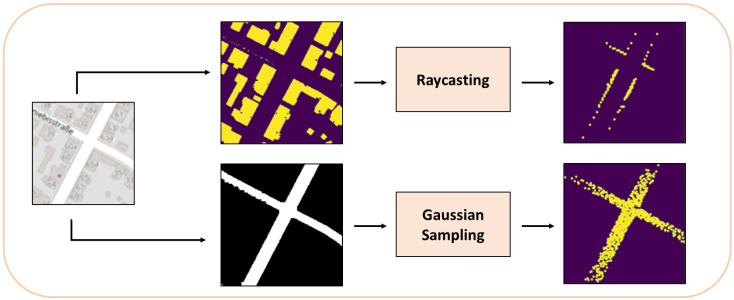
MPM. The OSM region of interest is segmented to produce a building and a road mask. Raycasting is applied to the building mask, whereas rejection sampling on the road mask with a Gaussian proposal is applied to the road mask, in order to produce two simulated top-view point cloud images.

For the simulated top section of the point cloud, we proceed to use raycasting on the building mask at the same position and region of interest used to generate the simulated road point cloud. Raycasting is a common method used to simulate LiDAR point clouds in autonomous driving cars simulators such as Carla [39]. By first detecting the obstacles in the surrounding environment (which are shown in the building mask in our case), we can use 2D raycasting and simulate a beam of light that stops when it hits an obstacle. By doing that in a 360-degree fashion, we are able to generate simulated LiDAR images. An example of this approach can be seen in Figure 5, and its results are compared with the images from the LiDAR sensor in Figure 6. The MPM is illustrated in Figure 7.

**Figure 8:**
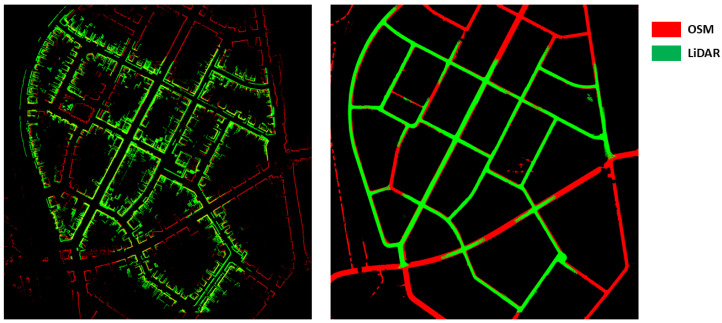
Comparison between OSM and LiDAR.

**Simulated LiDAR and OSM accuracy analysis.** In our case, providing an accurate localization result depends heavily on the accuracy of the map used. We start by building a 3D point cloud road map and a building map by using the ground truth odometry and segmentation provided by KITTI (note: this is only used here for visualization purposes and not during the rest of the manuscript), which we then proceed to project onto an image by using the previously discussed BEV projection. Following that, we also build a simulated LiDAR road map and building map from OSM by using color segmentatin and raycasting. By overlaying both maps from both modalities, we can see in Figure 8 that the maps generated by using OSM and raycasting are pretty accurate and match the maps created by using the LiDAR sensor. In come cases, occlusion of some buildings or the misrepresentation of road width on OSM can cause some mismatches; however, in the majority of cases, the shapes represented in both maps match.

### 3.2. Constrained Particle Filter

**Problem Formulation.** In order to localize our vehicle’s LiDAR in OSM, we use a constrained particle filter. In our configuration, we suppose that we have a guess of the initial pose of the vehicle in OSM (thanks to an external place recognition solution, or a GPS signal that was eventually lost) and each particle corresponds to a hypothetical position (x,y,α). The scale of the map is approximated by using the map size and its corner coordinates. After each LiDAR frame, we rely on our ICP based motion model to predict the particle positions by using two voxel-downsampled 3D LiDAR point clouds, and then update the particles weights by using results from the observation model. Low variance resampling [40] is triggered or not depending on the effective size of the particles [41], and finally the road check and subsequent constrained resampling is used if needed.

**Motion Model.** Our motion model relies on an ICP-based LiDAR odometry. We approximate the transformation between two subsequent LiDAR point clouds by using the Point-to-Point ICP algorithm [42]. We do not assume access to the vehicle controls, and only use the ICP output to update the particles positions.

**Observation Model.** Our observation model is the pre-processed input LiDAR point cloud, so we can match it to our simulated LiDAR images. As it was explained in the previous section, for each frame, we have access to four top-view images, two resulting from the LiDAR point cloud and two resulting from the simulated LiDAR, containing the road and the building edges in both modalities. Inspired by [20], we propose to calculate the weights of our particles based on combining the reciprocal chamfer distance between the pixel locations of the point clouds in each pair of images. The two terms forming the cost function are weighted by the reciprocal of the standard deviation of all the distances calculated above, as a form of uncertainty constraint on the weight’s distribution.

In summary, if $d_{c}$ is the chamfer distance, *N* the number of particles, $z_{r_{i}}$ and $z_{b_{i}}$ the list of all pixel positions of the points in the road and buildings LiDAR point clouds top-view images respectively, transformed according to the *i*th particle, and $m_{r}$ and $m_{b}$ the list of all pixel positions of the points in the top-view images of the simulated point clouds. We define $d_{r_{i}}=\frac{1}{d_{c}\left(z_{r_{i}},m_{r}\right)}$ and $d_{b_{i}}=\frac{1}{d_{c}\left(z_{b_{i}},m_{b}\right)}$, and calculate $\sigma_{r}$ and $\sigma_{b}$, which are the standard deviations of $d_{b}=\left\{d_{r_{1}},d_{r_{2}},\ldots ,d_{r_{N}}\right\}$ and $d_{r}=\left\{d_{b_{1}},d_{b_{2}},\ldots ,d_{b_{N}}\right\}$, respectively.

Finally, the weight of each *i*th particle can be defined as:

(1) $$\begin{array}{c} W_{i}=W_{ri}+W_{bi}=\frac{d_{r_{i}}}{\sigma_{r}}+\frac{d_{b_{i}}}{\sigma_{b}}. \end{array}$$

We choose to use the reciprocal of the chamfer distance in order to emphasize strong particle candidates that align the most with the map. The inclusion of the standard deviation terms is there to favor the data source that produce the most “concentrated” set of particles, which can be interpreted as being less uncertain about the final position. We attempted to calculate weights by using $exp(-d_{c}/\lambda )$ instead of $\frac{1}{d_{c}}$, but found the results to be very sensitive to the value of the parameter λ. We have also tested the use of a joint-probability formulation rather than the summed one, but saw no major difference in the final results.

**Constrained resampling.** When trying to approximate the correct position of the vehicle, we try to enforce a simple constraint: the vehicle position should always be on the road. This is done in the particle filter through a resampling procedure, inspired by the methods proposed in [36,37], that aims to gently push the final weighted position toward the correct region (in our case, the road). This is done by enforcing the constraint on the weighted mean value of the particles, rather than the particles themselves.

After updating the weights of the particles, we check to see if the weighted mean vehicle position resulting from the particle filter is on the road. If it is the case, no intervention is needed. Otherwise, we discard one of the particles outside of the road and resample one from the most highly weighted area on the road. Typically, only a few resampling steps (less than 50) are needed to validate our road check. If we reach the maximum resampling iteration, we relax the sampling criteria for the next step of the particle filter. For speed purposes, when resampling good particles to replace bad ones, we use inductive sampling: meaning we resample from the particles that are already available to us. A global view of our constrained particle filter method for localization of LiDAR point clouds in OSM is presented in Algorithm 1 and Figure 2.
**Algorithm 1** LiDAR-OSM Constrained Particle Filter Localization.**Input:** Point cloud *P*, OSM Region of Interest *J*.**Output:** Vehicle Position (x,y,α) Particles←ParticleSampling(J)$\left(P_{r},P_{b}\right)\leftarrow LidarProcessingModule\left(P\right)$$\left(P_{r}^{\prime },P_{b}^{\prime }\right)\leftarrow MapProcessingModule\left(J\right)$$Weights\leftarrow UpdateWeights(P_{r},P_{b},P_{r}^{\prime },P_{b}^{\prime })$**If**$n_{effectiveSize}<\frac{N}{2}$**then**(Particles,Weights)←LowVarReSampling(Particles,Weights)(x,y,α)←Estimate(Particles,Weights) // Split the particles on and off the road$P_{r},W_{r},P_{nr},W_{nr}\leftarrow OnOffRoadSplit\left(Particles,Weights,J\right)$ // Constrained Resampling**while**(x,y)∉Road**and**$size(P_{nr})>0$$P_{nr},W_{nr}\leftarrow DeleteByIndex\left(P_{nr},W_{nr},argmin\left(W_{nr}\right)\right)$$P_{r},W_{r}\leftarrow InductiveSampling\left(P_{r},W_{r}\right)$$Particles\leftarrow Concatenate(P_{r},P_{nr})$$Weights\leftarrow Concatenate(W_{r},W_{nr})$(x,y,α)←Estimate(Particles,Weights) **return**(x,y,α)

## 4. Experiments

### 4.1. Dataset

We use the KITTI Odometry Benchmark [43] to test our method and compare to other cross-modal or road-constrained localization methods. The KITTI dataset was collected in Karlsruhe, Germany by using a VW stationwagon equipped with a variety of sensor modalities such as high-resolution color and grayscale stereo cameras, LiDAR sensor and a high-precision GPS/IMU inertial navigation system. The recorded scenarios are diverse, ranging from urban scenarios, to highways or rural areas. Similar to the existing publications, we use the sequences (00, 02, 05, 07, 08, 09, 10) to test our approach. The naming of the sequences used here follows the KITTI Odometry Benchmark references instead of those from the raw KITTI dataset.

### 4.2. Implementation Details

During our experiments, we set the following parameters. Before using ICP, the LiDAR point clouds are first downsampled by using Voxel Downsampling with a resolution of 0.1 m, and we only keep points within a 30-m distance of the car, leaving around 50 k points for each point cloud. In the LPM, and because the LiDAR sensor is placed above the vehicle’s roof, we define the LiDAR point cloud top section as any point with a positive elevation value, and the opposite for the bottom section. After the projection to the top view, and before calculating the cost of the particle filter, we drastically downsample the road and building point clouds by only keeping 1000 points by using random sampling. This number was determined through trial and error, by increasing the number of points, until no improvements in accuracy were visible. During testing, we extract tiles from OSM-sized (320,320) pixels and we use 100 particles to achieve pose tracking. Finally, if one of two simulated point clouds has less than 50 points, we drop that point cloud and only use the second as input to the cost function. This would typically happen in areas where no buildings are around (in the beginning of sequence 09 for example) and only the road can be used to localize our vehicle.

### 4.3. Results

**Figure 9:**
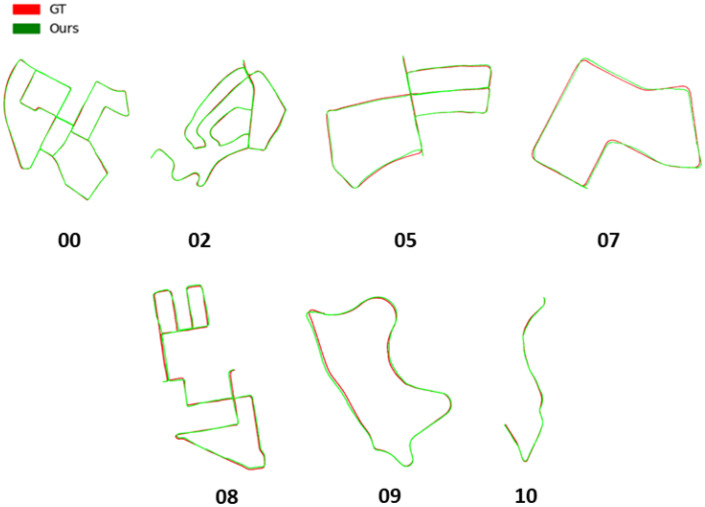
Qualitative results of our cross-modal pose tracking method on the KITTI dataset.

For context, we list the lengths of the tested KITTI sequences in Table 1. The results of our cross-modal localization approach, in comparison to other previously proposed methods in the literature are shown in Table 2 and Table 3. For both translation and rotation results, we use the accumulated mean error defined as $\frac{\sum_{i=1}^{N}\left|p_{i}-\bar{p_{i}}\right|}{N}$ to report our results, where $p_{i}$ and $\bar{p_{i}}$ are the predicted and ground truth poses, respectively, at the timestamp *i*. We compare our results to [16], which proposes a method to localize LiDAR point clouds in OSM by using handcrafted feature descriptors, [20] which proposes a method to localize LiDAR point clouds in satellite maps by using the correlation between the semantic segmentation of both modalities and [44], which presents a road-constrained monocular visual localization method. The results tagged [42] are those obtained when using the output of the ICP motion model only without the subsequent proposed observation model. Values marked N/A and rotation error values were not provided by the publications. Figure 9 shows a qualitative comparison between our final results and the ground truth.

In addition to the final accuracy that we achieve, if we take the length of the sequences into account, our method performs the best on sequence 00. This can be explained by the dense building layout and clear road boundaries of the area. On the other hand, the sequence where we struggle the most is sequence 08. The main reason behind that is the amount of trees and other foreign objects that are picked up in the LiDAR scan, which can produce noisy top-view images, thus leading the PF to wrong estimates. Fortunately, the constraint that we impose helps to keep the error bounded and makes it easier for the PF to correct itself later on.

### 4.4. Discussion

**Figure 10:**
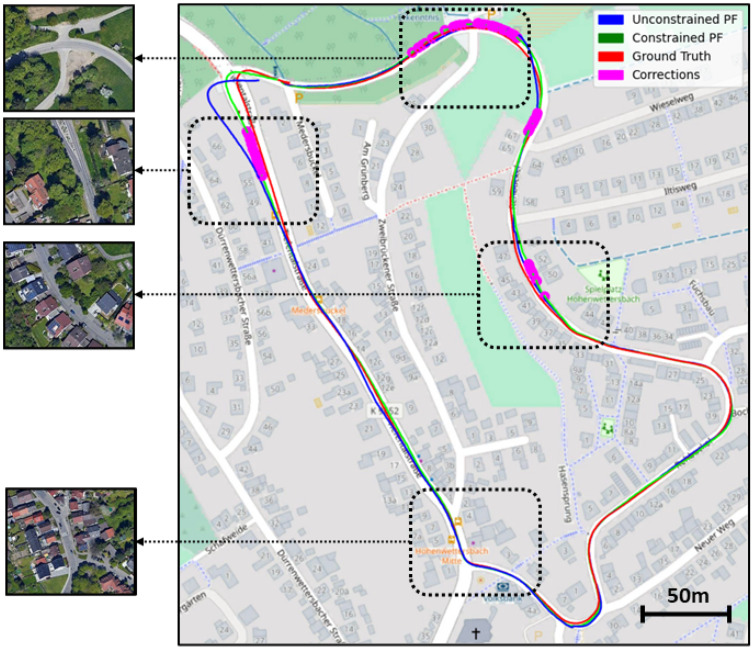
The effects of the constrained particle filter on sequence 09 of the KITTI dataset. Here, we show three cases where the constrained particle filter had to correct itself using the road structure, in addition to a case where it successfully estimated the right position using the output of the motion and observation models only.

**Constrained Particle Filter.** As stated above, the main goal of the constrained particle filter is to make sure that our final vehicle location is always on the road. We typically need to enforce this condition when the sensor data collected by the LiDAR is too noisy for the PF to produce accurate results. For example, in Figure 10, we show in purple the areas where the road constraint was enforced and the position corrected by the particle filter. This happens in areas where foreign objects such as tree trunks and thick vegetation are occluding the buildings, or when an intersection structure is not correctly represented on OSM, causing the particle filter to be misled.

Figure 10 also shows that our method does not rely on the constraint all the time and/or only tries to stay within the limits of the road. Instead, we are able to track our position correctly most of time by using the input data, and only trigger the constrained resampling in some corner cases, i.e., LiDAR scans are blocked by trees. As an additional example of a good case scenario, we show a region where the localization was successful, which can be explained by the fact that no major occlusion is occurring in that area, and that most structures present on and around the road are correctly represented in OSM.

Finally in Table 4, we show results that demonstrate the superiority of our constrained PF formulation to the acceptance/rejection approach used in [34] by comparing the mean translation error over two sequences. The goal here is to compare two ways of applying the constraints in a particle filter: first, applying the constraints to the final estimation of the particle filter, and second, applying the constraints while drawing the hypothesis. The latter can lead to wrong estimations (especially when intersections are involved), which can result in discontinued trajectories or even total failure of the pose tracking system. In contrast, our approach gently pushes the estimated pose towards the road whenever it is triggered, thus producing a smoother trajectory estimation.

**Figure 11:**
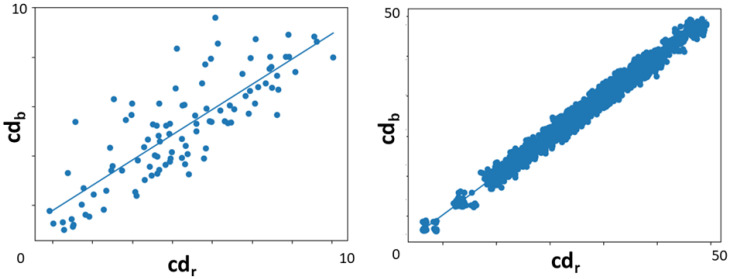
Chamfer distance correlation between road and building point clouds. On the **right**, mean distance values across sequence 05. On the **left**, distance values for a single random frame in sequence 00.

**Dual Input Particle Filter.** Our method uses both the road and building edges to come up with a guess at vehicle’s position by using the PF, preceding the road check validation. We use a standard deviation-based normalizer to give more value to the input, resulting in the best approximation. This is only safe to do if the chamfer distances calculated when using both inputs are correlated, which is verified in the KITTI dataset and demonstrated in Figure 11 for both a single random sample and across a full sequence.

**Figure 12:**
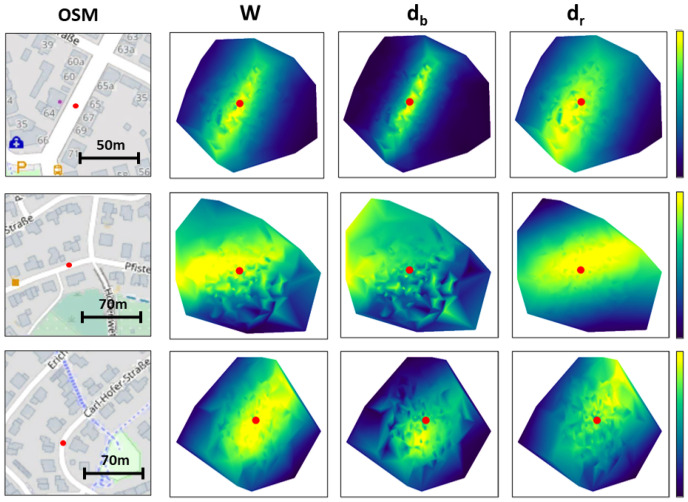
Interpolated heatmaps representing the weight distribution of the particles for different weights formulation, according to Equation (1). Red dots represent the true vehicle position.

In Figure 12, we show that it is necessary to use both inputs in order to get the best results possible. Figure 12 shows a list of heatmaps for different cases and the use of different weight formulations. We can see from the first case that of the weights resulting from the building edges, only $d_{b}$ tend to result in a coarse distribution, whereas for the road only $d_{r}$ tend to be smoother. This shows that both sets of weights, when combined, can be complimentary and result in a better final weighted sum position. While the heatmaps of the weights resulting from the buildings only in the first case could be interpreted as “more accurate” than the ones produced by the road, the second case that we show presents a situation where the buildings are occluded, resulting in noisy set of weights. If we only had access to these weights, then our position estimation would be way off compared to the results, we would get by combining both sets of weights, because the weights resulting from the road point cloud are reasonable in most cases. Finally, we show a case where both inputs result in distributions that are slightly off, whereas their combination is much more centered around the vehicle’s true position.

Note that it is also very useful to have access to both, in areas where only one of them is available: For example, at the beginning of sequence 09, no buildings are available and only the road can be used, as can be seen in top area of Figure 10.

**Figure 13:**
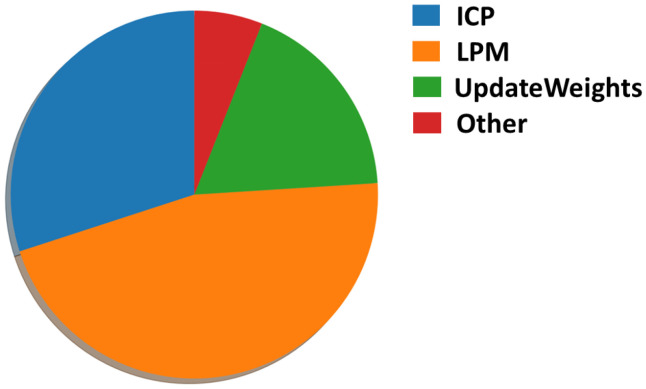
Runtime distribution.

**Runtime analysis.** Our method runs at a maximum speed of 10 Hz. The LPM takes the most amount of time with more than forty percent of the total runtime, followed by ICP with thirty percent and the UpdateWeights step with a little bit less than twenty percent. The constrained resampling, when triggered, consumes less than one percent of the total runtime on average. Figure 13 shows the runtime distribution for each part of the algorithm.

**Robustness.** We apply voxel-downsampling to all the sensor inputs in order to speed up the whole pipeline. Table 5 shows a comparison of the results of our method on sequences 07 and 10 when using different voxel sizes. These results show that the voxel size has little effect on the accuracy of our method, giving it the potential to work with LiDARs that produce sparser point clouds than the ones used in this study.

We also show a comparison in Table 6 between the use of random sampling and voxel sampling. Our results show that voxel downsampling is superior, which can be explained by its ability to conserve the general structure of the point clouds. In contrast, random sampling can sometime return wildly different point clouds from one frame to the other, leading to a decrease in accuracy when using ICP as a motion model, but it also can end up selecting most its points from a noisy or occluded area, which can negatively affect the accuracy of the particle filter.

## 5. Conclusions

This work proposes a general non-learning method for vehicle localization by using LiDAR and OSM. We first validate the accuracy level of the OSM data needed for vehicle localization. Subsequently, buildings and road masks are extracted to generate simulated LiDAR images by using raycasting. These images are compared with the BEV projection of the input LiDAR point cloud by using particle filters. In addition, the extracted road mask is applied as a constraint of the vehicle location, by making sure that the output location of our system is on the road, thus keeping our location error bounded. The evaluation results on KITTI show that the proposed method outperforms other existing cross-modal and road-constrained localization methods that were based on OSM and/or satellite maps. We also provide a detailed technical discussion and ablation studies to explain the advantages of the proposed method and demonstrate its ability to produce accurate pose tracking results. Future work will include a better way of dealing with occlusions in the sensor data, in addition to sensor fusion methods for more precise localization.

## Figures and Tables

**Table 1:** Comparison of the lengths of each of the tested KITTI sequences.

Sequences	00	02	05	07	08	09	10
Lengths (kms)	3.72	5.06	2.20	0.8	3.21	1.70	1.5

**Table 2:** Comparison of the translation error on KITTI dataset in meters (m). Best results are in bold.

Sequences	[20]		[16]		[44]		[42]		Ours	
	**Mean**	**Max**	**Mean**	**Max**	**Mean**	**Max**	**Mean**	**Max**	**Mean**	**Max**
00	2.0	12.0	20	150	3.7	14.01	51	162.2	**1.37**	**3.34**
02	9.1	35	N/A	N/A	11.32	25.6	114	307	**3.37**	**7.55**
05	N/A	N/A	25	150	4.02	8.7	20.75	70.34	**1.45**	**3.38**
07	N/A	N/A	25	120	N/A	N/A	7.07	15.47	**1.62**	**3.50**
08	N/A	N/A	N/A	N/A	4.67	11.96	62.99	195.22	**3.60**	**7.73**
09	7.2	20	25	50	5.72	11.57	35.5	96.2	**2.88**	**6.85**
10	N/A	N/A	180	50	N/A	N/A	14.73	29.6	**1.56**	**3.32**

**Table 3:** Comparison of the mean rotation error on KITTI dataset in degrees (°). Best results are in bold.

Sequences	00	02	05	07	08	09	10
[42]	58.5	40.2	9.3	13.2	44.3	20.7	6.9
Ours	**1.15**	**3.50**	**2.3**	**1.97**	**3.28**	**2.68**	**2.19**

**Table 4:** Constraint particle filter mean translation error (m) comparison.

Method	Sequence 05	Sequence 09
Ours	1.4	2.8
Acceptance/Rejection	17.8	3.2

**Table 5:** Voxel-downsampling resolution and mean translation (m) error comparison.

Voxel Resolution (m)	Sequence 07	Sequence 10
0.01	1.5	1.6
0.1	1.6	1.5
1	1.4	1.7

**Table 6:** Voxel-downsampling and random sampling mean translation error (m) comparison.

Sampling Method	Sequence 00	Sequence 07	Sequence 09
Random Sampling	2.53	3.59	9.69
Voxel Sampling	1.37	1.62	2.88

## Data Availability

The data that support the findings of this study are available from the KITTI dataset by the Karlsruhe Institute of Technology and the OpenStreetMap platform.
